# Electrophysiological Correlates of Emotional Source Memory in High-Trait-Anxiety Individuals

**DOI:** 10.3389/fpsyg.2016.01039

**Published:** 2016-07-12

**Authors:** Lixia Cui, Guangyuan Shi, Fan He, Qin Zhang, Tian P. S. Oei, Chunyan Guo

**Affiliations:** ^1^Beijing Key Laboratory of Learning and Cognition and Department of Psychology, Capital Normal UniversityBeijing, China; ^2^Psychological Health Education and Consultation Center, Dalian University of TechnologyDalian, China; ^3^Guanghua School of Management, Peking UniversityBeijing, China; ^4^School of Psychology, University of Queensland, BrisbaneQLD, Australia; ^5^James Cook UniversitySingapore, Singapore; ^6^Asia UniversityTaichung, Taiwan

**Keywords:** anxiety, source memory, old/new effect, emotion effect

## Abstract

The interaction between recognition memory and emotion has become a research hotspot in recent years. Dual process theory posits that familiarity and recollection are two separate processes contributing to recognition memory, but further experimental evidence is needed. The present study explored the emotional context effects on successful and unsuccessful source retrieval amongst 15 high-trait-anxiety college students by using event-related potentials (ERPs) measurement. During study, a happy, fearful, or neutral face picture first was displayed, then a Chinese word was superimposed centrally on the picture and subjects were asked to remember the word and the corresponding type of picture. During the test participants were instructed to press one of four buttons to indicate whether the displayed word was an old or new word. And then, for the old word, indicate whether it had been shown with a fearful, happy, or neutral face during the study. ERPs were generally more positive for remembered words than for new words and the ERP difference was termed as an old/new effect. It was found that, for successful source retrieval (it meant both the item and the source were remembered accurately) between 500 and 700 ms (corresponding to a late positive component, LPC), there were significant old/new effects in all contexts. However, for unsuccessful source retrieval (it meant the correct recognition of old items matched with incorrect source attribution), there were no significant old/new effects in happy and neutral contexts, though significant old/new effects were observed in the fearful context. Between 700 and 1200 ms (corresponding to a late slow wave, LSW), there were significant old/new effects for successful source retrieval in happy and neutral contexts. However, in the fearful context, the old/new effects were reversed, ERPs were more negative for successful source retrieval compared to correct rejections. Moreover, there were significant emotion effects for successful source retrieval at this time window. Further analysis showed ERPs of old items were more negative in fearful context than in neutral context. The results showed that early unsuccessful fearful source retrieval processes (related to familiarity) were enhanced, but late successful fearful source retrieval processes during source retrieval monitoring (related to recollection) were weakened. This provided preliminary evidence for the dual processing theory.

## Introduction

Episodic memory, referring to memory for an event or episode that occurs at a certain time and a certain place, includes two elements: item memory and source memory (Slotnick et al., 2003). Item memory refers to the recognition or recall of previously presented information itself, whereas source memory refers to the recollection or recall of the context from which the fact or information is acquired (Johnson et al., 1993). According to dual-process theories, when both item and source are remembered accurately, it is inferred that the memory decision is based on recollection. When the correct recognition of old items matches with incorrect source attribution, dual-process theories posit that the memory decision is based on familiarity. Familiarity and recollection are distinct cognitive processes. Familiarity relies on automatic processes and recollection relies on intentional processes.

The past studies have shown that the background emotional valence affects the retrieval of source memory, and we can speculate that both early item retrieval and late sources retrieval are affected by it (Jaeger et al., 2009). Some studies demonstrated that event-related potentials (ERPs) which were related to emotion-laden pictorial contexts as elicited during object recognition differed in two ways (Jaeger and Rugg, 2012). First, there was a relatively early-onset (circa 300–500 ms) positivity for objects encoded in emotional rather than in neutral contexts. Secondly, there was a positively inclined shift in the ERPs for emotionally encoded objects, which showed a relatively late onset (circa 700 ms). This was frontally distributed, and it persisted for several 100 ms. Findings from Smith et al. (2004) were replicated in Jaeger et al. (2009), by which the latter study team contrasted ERPs that had been studied in association with emotionally negative to neutral contexts during item recognition after the shorter study-test delay (10 min).

Past studies employing normal controls have demonstrated the effects of emotional contexts on memory retrieval (Smith et al., 2004; Jaeger et al., 2009; Jaeger and Rugg, 2012), but whether the emotion effects on successful retrieval are different from unsuccessful source retrieval has not been explored. One study showed that the emotion effects on information retrieval were different at different stages amongst high-trait anxiety individuals (Williams et al., 1988). One of the most elaborate theories for explaining selective information processing in anxiety was developed by Williams et al. (1988). This theory proposed that there should be two different stages of processing (i.e., automatic and strategic) related to the two distinct pathways of information integration and elaboration. At the integration stage of processing, if a stimulus is evaluated as threatening, people with high-trait-anxiety are likely to allocate cognitive resources to this new threatening information, thus, establishing a preference for processing this stimulus. In contrast, at the elaborative stage, there might not be extra resources allocated for cognitive processing, or cognitive resources for processing threatening stimuli may be removed altogether. This way, anxiety will either not be related to an enhanced recall of threatening stimuli (as when additional resources are unavailable) or be related to a worst memory of the threatening stimuli (as when baseline cognitive resources for stimuli processing are unavailable). The two processes are independent, meaning that a bias in one process is not associated with a bias in the other. Studying ERP differences produced in response to successful or unsuccessful source retrieval using different emotional contexts in high-trait-anxiety individuals would contribute to a deeper understanding of the relation between familiarity and recollection.

In the present study, we explored the cognitive and neural mechanisms of source retrieval under different emotional contexts in high-trait-anxiety individuals by using ERPs measurements and a source memory multiple-task paradigm. There were four responses in the test phase: (1) old item- happy context, (2) old item – fearful context, (3) old item – neutral context, and (4) new item. In the test-phase, trials for old items would be assigned to the condition of correct source retrieval (w/source) if the item was endorsed as an old item with the correct emotional context judgment, and if the item was endorsed as an old item but with the incorrect emotional context judgment these trials would be assigned to the condition of false source retrieval (w/o source). Using this paradigm we were able to compare how retrieval processing was moderated by the emotional context when the source retrieval was successful or unsuccessful. Based on the Dual-process theories (Williams et al., 1988), we hypothesized that the effects of emotional context on information retrieval differed depending on successful or unsuccessful source retrieval at different stages in high-trait-anxiety individuals.

## Methods

### Participants

Initially, 99 participants were recruited through advertising from several universities in Beijing. They also completed the State-trait Anxiety Inventory (STAI) and the top 30% of the STAI was selected for the study. Sixteen right-handed young adults were employed as subjects and remunerated at the rate of ¥20/hr. All study participants reported good health with no history of neurological or psychiatric illnesses. One participant with less than 16 artifact-free trials in at least one relevant condition was excluded from the analysis, leaving a final sample of fifteen participants (mean age was 21.81, *SD* = 1.59). Of the 15 subjects contributing data, 8 were women. The mean trait anxiety score for this group was 59.06 ± 3.88. Each participant signed an informed consent. This study was approved by the Institutional Review Board of the Capital Normal University.

## Materials

### State-Trait Anxiety Inventory (STAI)

The State-trait Anxiety Inventory (Speilberger et al., 1983) was utilized as the measure of anxiety symptoms. For the 20 trait items in the STAI, the students were asked to circle the number that best described how they generally felt with a four-point scale ranging from: 1 = almost never to 4 = almost always). All anxiety-absent (positively worded) items were reverse scored (i.e., reversed score = 5-original score). The internal consistency reliability (Cronbach’s α) of the Chinese versions of STAI was 0.88 and the Chinese undergraduates norm score of trait anxiety is 43.31 ± 9.20 (Li and Qian, 1995).

### Stimuli

In total, 1056 Chinese words were selected from Modern Chinese Frequency Dictionary and the average frequency of all these words is 25.0 occurrences/million (range = 4–82 occurrences/million) (Beijing Language and Culture University, 1986), of which 576 words were presented as old words both in the study and the test phase. Only 384 words were presented as new words in the test phase and 96 words were presented as filters in the study phase. The 1056 Chinese words were also rated on a nine-point scale by 50 college students in two dimensions: emotional valence and arousal. The results showed that the average emotional valence score of every word was 4.0 ± 0.56 and the average arousal score of every word was 4.0 ± 0.22.

**FIGURE 1:**
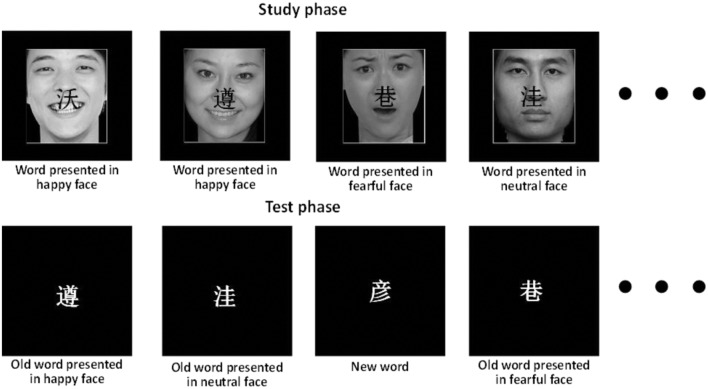
**Experimental stimulus sample**.

A total of 120 face pictures were drawn from the native Chinese Facial Affective Picture System (Wang and Luo, 2005), including 40 pictures of happy, fearful, and neutral face emotions, respectively. Pictures were presented within a white box to clearly demarcate their separation from the background. Eight pictures for each emotion were selected for the filters and practice. The rest of 96 pictures were presented as the context for target words, and each picture was repeated 6 times. The valence means of 32 pictures (16 male faces, 16 female faces) for each emotion was for happy, *M* = 6.69 ± 0.33; for neutral, *M* = 4.89 ± 0.30; for fearful, *M* = 2.67 ± 0.41. The results of one-way ANOVA with repeated measures showed that valence means differed significantly among the three emotions, *F*(2,62) = 978.42, *p* < 0.001. The arousal means of each emotion picture was as follows: for happy, 6.15 ± 0.92; for neutral, 3.74 ± 0.51; for fearful, 5.93 ± 1.05. The results of one-way ANOVAs of arousal for three emotion pictures showed a main effect of condition (*p* < 0.001). A Tukey HSD test revealed that the arousal level of fearful or happy emotion pictures was significantly higher than that of the neutral emotion pictures (*p* < 0.05). No significant difference was revealed between conditions pertaining to the arousal of fearful and happy emotions. All the pictures were similar in size, context, spatial frequency, contrast grade, brightness, and other physical properties. The Chinese words and the emotion pictures were randomly matched in each block. See experimental stimulus samples in **Figure 1**.

### Procedure

The experiment was conducted in a soundproof room. Subjects were seated in a quiet room with their eyes approximately100 cm from a 17-in screen. All face pictures were presented in the center of the screen. The viewing angle was 8.02 × 9.19°.

Using a study-test paradigm, the research was divided into 16 blocks. Each block comprised a study phase, a distraction phase, and a test phase. In the experiment, subjects were instructed to watch the center of the screen, and to relax and control their blinking.

**FIGURE 2:**
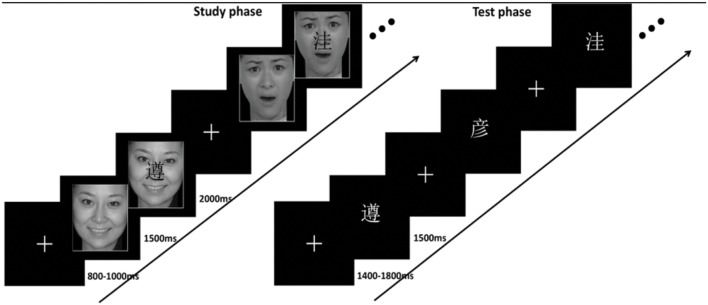
**Experimental paradigm.** During the study, a happy, fearful, or neutral face picture first was displayed, then a Chinese word was superimposed centrally on the picture and participants were asked to remember the word and the corresponding type of picture. During the test participants were instructed to press one of four buttons to indicate whether the displayed word was an old or new word. And then, for the old word, participants were instructed to indicate whether it had been shown with a fearful, happy, or neutral face.

The study phase included 42 trials, in which the first three and the last three trials were fillers. The other 36 trials, which were made up of 12 face pictures each for the three emotional contexts, and arranged pseudo-randomly with no more than three pictures from the same valence category presented consecutively. In one trial (see **Figure 2**), before presentation of the face picture, a fixation cross appeared at the central location during the inter stimulus interval (ISI) ranging from 800 to 1000 ms. Then the face pictures context was initially presented alone on the screen for 1500 ms. During this time subjects made a judgment on whether the face picture showed a fearful, happy, or neutral face. The responding fingers were balanced across participants. After presenting the context, the Chinese word was superimposed centrally on the face picture. Subjects were required to remember the word and the corresponding type of face picture (i.e., fearful/happy/neutral). The word and face picture were presented together for 2000 ms, after which the fixation cross appeared on screen, before the next face picture was presented. The emotion pictures were balanced for gender, and repeated for the same number of times. The Chinese words were not emotionally laden and not repeated in the study phase.

The test phase followed the study phase after a delay of around 1 min, during which a serial subtraction task was used to minimize rehearsal effects. In the test phase of the item memory task, each block consisted of 36 studied words, 6 filler items, and 24 new words, each of which was presented for 1500 ms with an ISI ranging from 1400 to 1800 ms. Each participant was instructed to press one of four buttons on the response box to indicate whether it was an old or new word. And then, for those that were old words, the participants were instructed to indicate whether it had been shown with a fearful, happy or neutral face (See **Figure 1**). The responding fingers were balanced across participants. To avoid key confusion, the buttons of emotion pictures in study phase corresponded with those in the test phase.

### ERP Recording and Analysis

Electroencephalographic recordings were obtained from 62 scalp sites using Ag/AgCl electrodes embedded in an elastic cap at locations from the extended International 10–20 System. These electrodes were referenced to the right mastoid during recording and re-referenced to the average of the right and left mastoid offline. Two additional channels were used for monitoring horizontal and vertical electrooculographic (EOG) recordings. Impedance was reduced below 5 KΩ. EEG signals were filtered with a band-pass of 0.05–40 Hz and sampled at a rate of 500 Hz. Each epoch lasted 1600 ms, including 200 ms prior to stimulus onset. Trials with a voltage, relative to the 200-ms baseline, exceeding ± 75 μV at any electrode were excluded from analysis, as were trials with artifacts in the EOG channels. ERPs were quantified by measuring mean amplitudes in three latency intervals (300–500, 500–700, and 700–1200 ms for the test phase), relative to the mean amplitude of the pre-stimulus baseline (-200–0 ms). These intervals were selected based on visual inspection of grand-average ERPs, given that similar intervals have been used in prior studies of related ERP phenomena. Although initial analyses focused on three midline locations (Fz/Cz//Pz), topographic analyses confirmed that these midline locations captured the most important effects. ERPs were averaged for test phase data, when words were presented with a blank background. In the test-phase, trials for old items were classified as hit _item w/source_ if the item was endorsed as an old item with the correct source judgment, and as hit _item w/o source_ if the item was endorsed as an old item but with the incorrect source judgment. The hit _item w/source_ and hit _item w/o source_ both were tested for three different emotional contexts. Meanwhile, new trials were classified as correct rejections, if they were correctly endorsed as new, and as false alarms, if incorrectly endorsed as old. The hit _item w/source_ and hit _item w/o source_ both have three types as for different emotion.

For each dependent variable, an ANOVA with repeated measures was performed. All ANOVAs were two-tailed with a level of significance set to α = 0.05 and supplemented with pairwise comparisons or simple effect comparisons when appropriate. For all effects with two or more degrees of freedom in the numerator, we adjusted when appropriate for violations of sphericity, which are inherent in ANOVAs, according to the Greenhouse and Geisser (1959) formula. Midline ERP measurements were evaluated using a condition-by-electrode-location ANOVA for each latency interval. Main effects of electrode location are not reported.

## Results

### Behavioral Results

Given the four-key response requirements in the source test, we analyzed two different hits in three different emotion contexts. One hit was correct item and source (hit _item w/source_). The other was when the source judgment was incorrect (hit _item w/o source_). The mean accuracy and reaction times (RTs) for hit _item w/source_ and hit _item w/o source_ in fearful, happy, and neutral contexts, as well as data for correctly rejected new items, are shown in **Table 1.**

**Table 1:** Means (and SEs) for the Accuracies and Reaction Times (RTs) for each condition.

	Old				New
		Fearful context	Happy context	Neutral context	(Correct rejection)
Accuracy (SE)	hit _item w/source_	0.52 (0.14)	0.58 (0.13)	0.55 (0.12)	0.78 (0.20)
	hit _item w/o source_	0.31 (0.14)	0.30 (0.10)	0.23 (0.09)	
Reaction time in ms (SE)	hit _item w/source_	1013.70 (121.32)	957.39 (113.52)	973.15 (118.53)	847.41 (74.47)
	hit _item w/o source_	1075.94 (170.58)	1044.18 (164.24)	1074.57 (176.43)	


*In the test phase, new trials were classified as correct rejections if correctly endorsed as new; old trials were classified as hit _item w/source_ if the item was endorsed as an old item with the correct source judgment, as hit _item w/o source_ if the item was endorsed as an old item but with the incorrect source judgment. The hit _item w/source_ and hit _item w/o source_ both have three types for different emotions.*

### Hit Rates

One-way repeated-measures ANOVAs were conducted on hit rates of the three conditions (hit _item w/source_, hit _item w/o source_, correct rejections) separately for three different emotion face contexts. A main effect of condition was observed for all of the three different emotion face contexts (*p*s < 0.001). Tukey HSD tests revealed that both hit _item w/source_ rates and hit _item w/source_ rates were lower than correct rejection rates, meanwhile hit _item w/source_ rates were higher than hit _item w/o source_ rates (*p*s < 0.05), regardless of the emotional context (See **Table 1**).

One-way repeated-measures ANOVAs were conducted on hit _item w/source_ rates with three different emotional contexts and no main effect of emotional context was observed (*p* > 0.05). But the results of one-way repeated ANOVAs conducted on hit _item w/o source_ rates with three different emotion contexts showed a main effect of emotional context, *F*(2,28) = 6.946, *p* < 0.01, η^2^ = 0.19. A Tukey HSD test revealed that hit _item w/o source_ rates, either in fearful or in happy context, were higher than hit _item w/o source_ rates in neutral context (*p*s < 0.05) (See **Table 1**).

### RTs

One-way repeated ANOVAs were conducted on RTs with three conditions (hit _item w/source_, hit _item w/o source_, correct rejections) separately for three different emotional contexts. A main effect of condition was observed for all the three emotional contexts (*p*s < 0.001). Tukey HSD tests revealed that both hit _item w/source_ RTs and hit _item w/source_ RTs were longer than correct rejections RTs regardless of the emotional context. On the other hand, hit _item w/source_ RTs were shorter than hit _item w/o source_ RTs in happy and neutral contexts (*p*s < 0.05), while there was no significant difference between hit _item w/source_ RTs and hit _item w/o source_ RTs in fearful context (*p* > 0.05).

One-way repeated-measures ANOVAs were conducted on RTs for hit _item w/source_ with three different emotional contexts and a main effect of emotion context was observed, *F*(2,28) = 8.262, *p* < 0.01, η^2^ = 0_._41. A Tukey HSD test revealed that RTs for hit _item w/source,_ either in neutral or happy context, were significantly faster than RTs for hit _item w/source_ in fearful context (*p*s < 0.05). No significant difference was revealed between RTs for hit _item w/source_ in neutral and happy context. One-way repeated ANOVAs conducted on RTs for hit _item w/o source_ with three different emotional contexts showed a main effect of emotional context, *F*(2,28) = 3.732, *p* < 0.05, η^2^ = 0.26. A Tukey HSD test revealed that RTs were significantly slower for hit _item w/o source_ in fearful context than in happy context (*p* < 0.05). No significant difference was found between RTs in fearful and neutral context (see **Table 1**).

### Event-Related Potential Data

The mean (range) number of trials contributing to the average ERPs for each response type were for correct rejections, 265 (126–370); for fear/hit _item w/source_, 93 (51–130); for fear/hit _item w/o source_, 51 (20–94); for happy/hit _item w/source_, 100(55–138); for happy/hit _item w/o source_, 51(26-91); for neutral/hit _item w/source_, 94(43–130); and for neutral/hit _item w/o source_, 39(20,71).

Test-phase ERPs were analyzed separately for each condition. Clear differences were observed beginning about 300 ms after stimulus onset and lasted for 900 ms. ERPs were generally more positive for remembered words than for new words. This ERP difference is termed as an old/new effect and the old/new effect as a measure of cognitive and neural mechanisms of retrieval has been widely used (Friedman and Johnson, 2000). **Figure 2** shows ERPs from the three chief conditions in the test phase. Two different old/new effects were computed. The first was based on correct source recognition, such that hit _item w/source_ trials were compared to correct rejections. The second old/new effect was based on recognizing the item as old but with the wrong context; hit _item w/o source_ trials were compared to correct rejections. We are most interested in the difference of these two old/new effects and the different emotion effects on them. Thus, first: two different old/new effects would be contrasted separately for three different emotion face pictures that followed. These repeated-measures ANOVAs with two factors, condition (correct rejections/hit _item w/source_/hit _item w/o source_) and electrode location (Fz/Cz/Pz), were conducted on mean amplitude data for three time intervals, 300–500 ms (corresponding to a negative-going wave), i.e., N400 ms, 500–700 ms (corresponding to a late positive component, i.e., LPC), and 700–1200 ms (corresponding to a late slow wave, i.e., LSW). These time window regions were selected based on the timing of memory effects in previous studies (Rugg and Allan, 2000; Maratos and Rugg, 2001; Smith et al., 2004) and visual inspection of the present data. Secondly, another repeated-measures ANOVA with two factors, emotion condition (i.e., fearful/happy/neutral) and electrode location (Fz/Cz/Pz), was conducted to compare emotion effects separately in conditions of hit _item w/source_ and hit _item w/o source_.

### Old/New effects

*In fearful context* The results of the ANOVA with two factors, condition (correct rejections/hit _item w/source_/hit _item w/o source)_ and electrode location (Fz//Cz//Pz) in the test phase of the 300–500 ms revealed a main effect of condition, *F*(2,13) = 39.120, *p* < 0.001, η^2^ = 0.33 and significant interaction effects between condition and electrode location, *F*(4,56) = 4.269, *p* = 0.03, η^2^ = 0.37. *Post hoc* comparisons (Bonferroni) showed the N400s were more positive both for fearful hit _item w/source_ and fearful hit _item w/o source_ compared to correct rejections (*p*s < 0.001) and there was no significant difference between hit _item w/source_ and hit _item w/o source._ (*p*s > 0.05).

**FIGURE 3:**
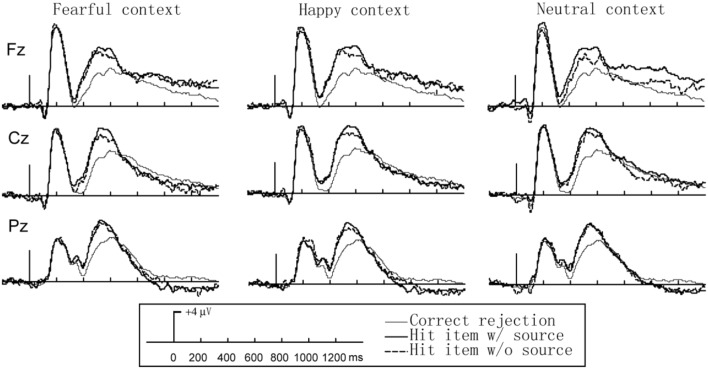
**Event-related potential (ERP) waveforms for hit _item w/source_, hit _item w/o source_, and correct rejection in the source test in different emotional valence contexts (Hit trials are correct responses to old items, and correct rejection trials are correct responses to new items)**.

ANOVA of the 500–700 ms latency region revealed a main effect of condition, *F*(2,13) = 8.978, *p* < 0.05, η^2^ = 0.42. *Post hoc* comparisons (Bonferroni) showed the LPCs were more positive for both fearful hit _item w/source_ and fearful hit _item w/o source_ compared to correct rejections (*p*s < 0.001) and there was no significant difference between hit _item w/source_ and hit _item w/o source_ (*p*s > 0.05; see **Figure 3**).

For the 700–1200 ms latency region, ANOVA of the data revealed an interaction between condition and electrode location, *F*(4,56) = 12.476, *p* < 0.001, η^2^ = 0.27. *Post hoc* comparisons (Bonferroni) showed that for the hit _item w/source_ old/new effect was reversed and the LSW amplitudes were larger for correct rejections than for fearful hit _item w/source_ at *P*z (*p* < 0.05). The hit _item w/o source_ old/new effect was not significant (*p*s > 0.05) and there was no significant difference between hit _item w/source_ and hit _item w/o source_ (*p*s > 0.05) (see **Figure 3**).

*In a happy context* The results of the ANOVA with two factors, condition (correct rejections/hit _item w/source_/hit _item w/o source_) and electrode location (Fz/Cz/Pz) in the test phase of the 300–500 ms revealed a main effect of condition, *F*(2,13) = 59.12, *p* < 0.001, η^2^ = 0.27 and significant interaction effects between condition and electrode location, *F*(4,56) = 4.269, *p* < 0.05, η^2^ = 0.29. *Post hoc* comparisons (Bonferroni) showed the N400s were more positive for both fearful hit _item w/source_ and fearful hit _item w/o source_ compared to correct rejections (*p*s < 0.001) and there was no significant difference between hit _item w/source_/and hit _item w/o source_ (*p*s > 0.05).

ANOVA of the 500–700 ms region revealed a main effect of condition, *F*(2,13) = 10.376, *p* < 0.001, η^2^ = 0.30 and significant interaction between condition and electrode location, *F*(4,56) = 3.778, *p* < 0.05, η^2^ = 0.36. *Post hoc* comparisons (Bonferroni) showed that the LPCs were more positive for happy hit _item w/source_ compared to correct rejections (*p*s < 0.001), but there were no significant difference between hit _item w/o source_ and correct rejections (*p*s > 0.05) and no significant differences between hit _item w/source_/and hit _item w/o source_ (*p*s > 0.05) (see **Figure 3**).

ANOVA of the 700–1200 ms revealed a significant interaction between condition and electrode location, *F*(4,56) = 10.900, *p* < 0.001, η^2^ = 0.36. *Post hoc* comparisons (Bonferroni) showed the LSWs were more positive for happy hit _item w/source_ compared to correct rejections at Fz (*p* = 0.037), but neither did for hit _item w/o source_ (*p*s > 0.05) and there was no significant difference between hit _item w/source_/ and hit _item w/o source_ (*p*s > 0.05) (see **Figure 3**).

*In a neutral context* The results of the ANOVA with two factors, condition (correct rejections/hit _item w/source_/hit _item w/o source_) and electrode location (Fz/Cz/Pz) in the test phase of the 300–500 ms revealed a main effect of condition, *F*(2,13) = 19.12, *p* < 0.001, η^2^ = 0.26 and significant interaction effects between condition and electrode location, *F*(4,56) = 4.269, *p* = 0.03, η^2^ = 0.17. *Post hoc* comparisons (Bonferroni) showed the N400s were more positive for both fearful hit _item w/source_ and fearful hit _item w/o source_ compared to correct rejections (*p*s < 0.001) and there was no significant difference between hit _item w/source_/and hit _item w/o source_ (*p*s > 0.05) (see **Figure 3**).

ANOVA of the 500–700 ms revealed a main effect of condition, *F*(2,13) = 13.059, *p* = 0.003, η^2^ = 0.36 and a significant interaction between condition and electrode location *F*(4,56) = 14.23, *p* = 0.016, η^2^ = 0.17. *Post hoc* comparisons (Bonferroni) showed that the LPCs were more positive for happy hit _item w/source_ compared to correct rejections in all electrode locations, *F*z (*p* = 0.04), Cz (*p* = 0.021), *P*z (*p* = 0.013), but there were no significant differences between hit _item w/o source_ and correct rejections (*p*s > 0.05) and no significant differences between hit _item w/source_ and hit _item w/o source_ (*p*s > 0.05) (see **Figure 3**).

For the 700–1200 ms, ANOVA of the data revealed an interaction between condition and electrode location, *F*(4,56) = 14.740, *p* < 0.001, η^2^ = 0.19. Further analysis showed the LSW amplitudes were larger for neutral/hit _item w/source_ than for correct rejections at *F*z (*p* = 0.004), but neither did for hit _item w/o source_ (*p*s > 0.05) and there was no significant difference between hit _item w/source_/and hit _item w/o source_ (*p*s > 0.05) (see **Figure 3**).

### Emotion Effects

To compare the effects of emotion on the ERPs elicited by hit _item w/source_ in three different emotional contexts, repeated-measures ANOVA with two factors, i.e., emotional context (fear/happy/neutral) and electrode location (Fz/Cz/Pz) was conducted on mean amplitude data for three time intervals, 300–500, 500–700, and 700–1200 ms. The repeated-measures ANOVA results showed no significant difference on mean amplitude data for two time intervals, 300–500 and 500–700 ms. The ANOVA results of the 700–1200 ms revealed a main effect of condition *F*(2,28) = 4.086, *p* = 0.028, η^2^ = 0.19. *Post hoc* comparisons (Bonferroni) showed that the LSWs were more negative for fearful hit _item w/source_ as compared to neutral hit _item w/source_ and no significant difference between neutral and positive context or between negative and positive context (*p*s > 0.05).

**FIGURE 4:**
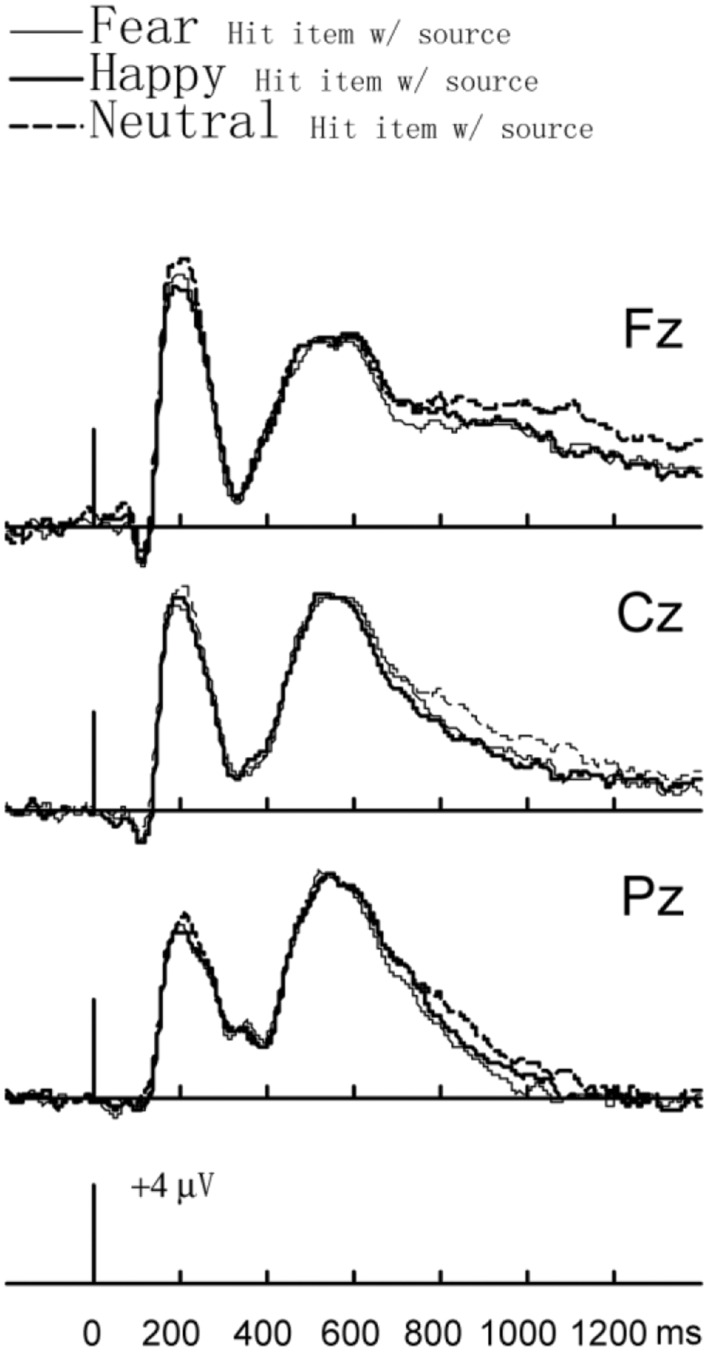
**Event-related potential waveforms for fear hit _item w/source_, happy hit _item w/source_, and neutral hit _item w/source_ in the source test**.

Similarly, the repeated-measures ANOVA of hit _item w/o source_ with two factors, i.e., emotion context (fear/happy/neutral) and electrode location (Fz/Cz//Pz), showed no significant difference on mean amplitude data for three time intervals, 300–500, 500–700, and 700–1200 ms (*p*s > 0.05) (see **Figure 4**).

## Discussion

By using a source memory multiple-task paradigm this study explored the differences between successful source retrieval, unsuccessful source retrieval, and different emotional context effects on them. During the test phase, correct rejections need only item retrieval, but successful or unsuccessful source retrieval needs not only item retrieval but also source retrieval. So compared with correct rejections, successful and unsuccessful source retrieval were difficult. Our behavioral data suggested that hits of successful and unsuccessful source retrievals were lower and their RTs were longer than that of correct rejections. This was consistent with a previous study demonstrating that source retrieval was later than item retrieval (Guo et al., 2006). And compared with successful source retrieval, the hit of unsuccessful source retrieval was lower and the RTs of unsuccessful source retrieval were longer. This was consistent with previous findings. Results from this study suggest that RT could be extended either by unsuccessful source retrieval or uncertain source retrieval (Cansino et al., 2002). However, only in the fearful context, hit _item w/o source_ RTs were not observed to differ significantly from hit _item w/source_ RTs. There is a possibility that highly anxious individuals experience some form of interference from fearful stimuli even when correct judgments have been made.

As for emotional effects, hit _item w/source_ rates did not show significant differences among different emotional contexts. On the other hand, hit _item w/source_ RTs were significantly longer in fearful than in neutral and happy contexts. Hit _item w/o source_ rates in happy and fearful contexts were higher than that in the neutral context. For hit _item w/o source_ RTs, there was no significant difference between fearful and neutral contexts. However, hit _item w/o source_ RTs were significantly longer in the fearful than in the happy context. The higher hit _item w/o source_ rates in emotional contexts suggested that emotional contexts may facilitate the unsuccessful source retrieval in highly anxious individuals. The longer hit _item w/source_ RTs in fearful context suggested that highly anxious individuals may spend more time making judgments in fearful contexts during successful source retrieval.

Consistent with many prior reports, ERPs of successful and unsuccessful source retrieval were more positive than new items in the three different emotion contexts between 300 and 500 ms after stimulus onset (old/new effect; Mecklinger, 2000; Curran and Cleary, 2003; Speer and Curran, 2007). In addition, there was no difference between the old/new effects of hit _item w/source_ and hit _item w/o source_ conditions tested at this window in the three different emotional contexts. This suggested that N400 was related to early item retrieval and independent of source retrieval. Moreover, the current study indicated that item retrieval during 300–500 ms was not influenced by emotional contexts. It seemed that item familiarity had not been modulated by the nature of the emotional context.

In the 500–700 ms (LPC), hit item_w/source_ old/new effects existed in all electrode locations in the three emotional contexts. One effect relevant to the present study is a positivity toward a left parietal maximum, i.e., the so-called left parietal old/new effect. This effect goes into onset around 400 ms post-stimulus, has a duration of around 500 ms, and is thought be a correlate of episodic retrieval or “recollection” (Smith, 1993; Rugg et al., 1996). A second relevant effect is the “right frontal old/new effect.” As implied by name, this is maximal over the right frontal scalp and goes into onset around 500–600 ms post stimulus, persisting for a second or more. The effect has been proposed as a neural correlate of post retrieval monitoring (Wilding and Rugg, 1996; Rugg et al., 1998). Some studies also found that brain regions associated with recollection were distributed closer to the frontal-central area (Duarte et al., 2004; MacKenzie and Donaldson, 2007; Speer and Curran, 2007). These showed that the distribution of the source memory covers a wide range of brain regions, including frontal, frontal-central area, and parietal area. They might have different functions in the retrieval on source memory. Supporting this, results in this study revealed non-significant emotion effects for hit _item w/source_ condition when tested among the three emotion contexts at this window. All these results seemed to show that successful source retrieval (recollection) had not been modulated by the emotional context during 500–700 ms in the high-trait-anxiety individuals.

The ERPs for hit _item w/o source_ in the fearful context were more positive than that for correct rejections during 500–700 ms time window. No such difference was revealed in the happy and neutral context. Thus, the higher positivity for fearful hit _item w/o source_ compared with correct rejections may suggest that the high-trait-anxiety participants might have an enhanced memory bias for fearful source information related to source familiarity. According to dual process theory familiarity relies on automatic processes. This is consistent with the theory of selective information processing by Williams et al. (1988), which posited that highly anxious individuals display a memory bias for negative stimuli at the integration (automatic) stage of processing.

In the 700–1200 ms time window (LSW), hit _item w/source_ old/new effects were observed at F_Z_ electrode site and ERPs of hit _item w/source_ in happy and neutral contexts were more positive than that of correct rejections. However, ERPs for hit _item w/source_ in electrode P_Z_ in the fearful context were more negative than that for correct rejections. Additionally, emotion effect analyses showed that at the 700–1200 ms time windows, ERPs of hit _item w/source_ in the fearful context were more negative than that of hit _item w/source_ in the neutral context. This result, together with the result of reversed hit _item w/source_ old/new effects in the fearful context in the 700–1200 ms time windows, implied that the high-trait-anxiety participants minimized an explicit recollection of fearful information during source retrieval monitoring. Our findings were consistent with prior studies, one of which revealed that the slow waves at the left parietal area for anxious individuals were smaller for negative stimuli compared to other types of stimuli (Inaba and Ohira, 2009). A possibility was the result of problems in attention among high-trait- anxiety participants (Williams et al., 1997). It has been suggested that anxious individuals avoid the elaborative processing of negative stimuli (Mogg et al., 1989; Holmes et al., 2008). For example, Mogg et al. (1989) reported greater interference in a Stroop task and lower accuracy for negative items in a subsequent recognition memory task among high-anxiety individuals. These findings were inferred to be due to selective attention and an avoidance of elaborative encoding of negative items. Recently, Holmes et al. (2008) reported that when looking at a fearful face, the high-trait-anxiety group showed increased visual P1 and less significantly enhanced fronto-central positivity and EPN as compared with the low-trait-anxiety group. Considering all these results above, anxiety may favor attention processing mechanisms at early stages over the effort- and time-consuming elaborative processing mechanisms, due to the major role that anxiety plays in the detection and avoidance of negative or dangerous encounters in one’s surroundings (Mogg and Bradley, 1998).

However, one limitation of the present study was that only the high trait-anxiety-group was used and, thus, it was unable to say whether the findings were actually driven by trait anxiety. A normal control or low trait anxiety group would be needed for such a purpose. Another limitation was that the sample size was rather low. Although we had sufficient power to detect the influences of fearful context based *p*-values and effect sizes provided in the results section, future studies with larger sample size are still needed to help support the conclusion made in the present study.

## Conclusion

This study provided preliminary behavioral and electrophysiological evidence of the effects of emotion context on source memory processing mechanisms in high-trait-anxiety participants. Study results showed that early unsuccessful fearful source retrieval processes (related to source familiarity) were enhanced, but late successful fearful source retrieval processes during source retrieval monitoring (related to source recollection) were weakened in high anxiety individuals. However, in happy and neutral contexts, results differed. This study provided preliminary evidence for the dual processing theory.

## Author Contributions

LC and GS the main writers and contributors of the paper and contributed equally to this work. CG the director of the research. GS completed most parts of the experiment and data analysis. FH finished part of the data analysis. GS and ZQ revised the paper. TO finished the English editing.

## Conflict of Interest Statement

The authors declare that the research was conducted in the absence of any commercial or financial relationships that could be construed as a potential conflict of interest.
